# Copper metabolism-related risk score identifies hepatocellular carcinoma subtypes and SLC27A5 as a potential regulator of cuproptosis

**DOI:** 10.18632/aging.205334

**Published:** 2023-12-28

**Authors:** Xiaoyan Li, Jinping Wang, Zongliang Guo, Yong Ma, Dawei Xu, Daguang Fan, Peng Dai, Yifan Chen, Qiongwen Liu, Jinke Jiao, Jinhan Fan, Ningxue Wu, Xin Li, Guoyin Li

**Affiliations:** 1 Department of Blood Transfusion, Shanxi Provincial People’s Hospital, Affiliate of Shanxi Medical University, Taiyuan, Shanxi, China; 2 Department of Central Laboratory, Shanxi Provincial People's Hospital, Affiliate of Shanxi Medical University, Taiyuan, Shanxi, China; 3 Department of Ultrasound, Shanxi Provincial People's Hospital, Affiliate of Shanxi Medical University, Taiyuan, Shanxi, China; 4 Department of General Surgery, Shanxi Province Cancer Hospital, Affiliated of Shanxi Medical University, Taiyuan, Shanxi, China; 5 Department of Thoracic Surgery, Shanxi Province Cancer Hospital, Affiliated of Shanxi Medical University, Taiyuan, Shanxi, China; 6 Department of Hepatobiliary and Pancreatic Surgery, Shanxi Provincial People's Hospital, Affiliate of Shanxi Medical University, Taiyuan, Shanxi, China; 7 College of Management, Zhejiang Shuren University, Hangzhou, Zhejiang, China; 8 College of Life Science and Agronomy, Zhoukou Normal University, Zhoukou, Henan, China; 9 Department of Geriatric Medicine, Shanxi Provincial People's Hospital, Affiliate of Shanxi Medical University, Taiyuan, Shanxi, China; 10 MOE Key Laboratory of Modern Teaching Technology, Center for Teacher Professional Ability Development, Shaanxi Normal University, Xi’an, Shannxi, China; 11 Academy of Medical Science, Zhengzhou University, Zhengzhou, Henan, China

**Keywords:** copper metabolism, cuproptosis, hepatocellular carcinoma, immunotherapy, SLC27A5

## Abstract

Aims: Dysregulated copper metabolism has been noticed in many types of cancer including hepatocellular carcinoma (HCC); however, a comprehensive understanding about this dysregulation still remains unclear in HCC.

Methods: A set of bioinformatic tools was integrated to analyze the expression and prognostic significance of copper metabolism-related genes. A related risk score, termed as CMscore, was developed via univariate Cox regression, least absolute shrinkage and selection operator (LASSO) Cox regression and multivariate Cox regression. Pathway enrichment analyses and tumor immune cell infiltration were further investigated in CMscore stratified HCC patients. Weighted correlation network analysis (WGCNA) was used to identify potential regulator of cuproptosis.

Results: Copper metabolism was dysregulated in HCC. HCC patients in the high-CMscore group showed a significantly lower overall survival (OS) and enriched in most cancer-related pathways. Besides, HCC patients with high CMscore had higher expression of pro-tumor immune infiltrates and immune checkpoints. Moreover, cancer patients with high CMscore from two large cohorts exhibited significantly prolonged survival time after immunotherapy. WGCNA and subsequently correlation analysis revealed that SLC27A5 might be a potential regulator of cuproptosis in HCC. *In vitro* experiments revealed that SLC27A5 inhibited cell proliferation and migration of HCC cells and could upregulate FDX1, the key regulator of cuproptosis.

Significance: The CMscore is helpful in clustering HCC patients with distinct prognosis, gene mutation signatures, and sensitivity to immunotherapy. SLC27A5 might serve as a potential target in the induction of cuproptosis in HCC.

## INTRODUCTION

Copper (Cu) participates in several important biological processes, including oxygen metabolism, iron homeostasis, synthesis of neurotransmitters and antioxidant defense, as a result, it is essential for the survival of our human body [1–3]. A bunch of studies have demonstrated that copper dyshomeostasis, either excess level of copper or deficiency of the ion, is harmful and results in various types of disease [2]. Wilson’s disease is a typical one due to overload of copper caused by pathogenic mutations in the ATP7B gene encoding ATP-ase7B, which is ineffective in transporting copper out of the cell [4, 5]. Copper also plays a role in promoting Parkinson’s disease, in which copper might lead to accumulation of amyloid fibril, a major component of Lewy bodies [6–8].

The dysregulation of Cu homeostasis in cancer has also been well documented [9–11]. As a key regulator of many signaling pathways and component in various essential enzymes, copper ion is supposed to be a promoter in the carcinogenesis and advancement of multiple types of tumors [9, 12]. Elevated level of copper ion is found in a variety of cancer such as breast cancer, lung cancer, bladder cancer, and head and neck cancer [13–16]. And copper ion could support tumor cell proliferation, metastasis, and angiogenesis [9, 17]. High level of serum copper has also been linked to worse prognosis in some types of cancer like hepatocellular carcinoma (HCC) and breast cancer [18, 19]. Besides, many basic researches and some early-stage clinical trials demonstrated that copper chelators, decreasing Cu concentration in tumor cells, had anti-tumor efficiency [17, 20, 21]. However, elevating Cu concentration in malignant tumors can also produce anti-cancer effect partially due to oxidative stress triggered by excess copper ions [17]. Correspondingly, Cu-containing compounds or Cu ionophores, like disulfiram (DSF) and dithiocarbamates, showed tumor-killing effects both *in vivo* and *in vitro* [17, 22]. In particular, a novel copper-induced cell death, distinct from previously described ways of cell death like apoptosis, necroptosis, and ferroptosis, has been discovered and termed cuproptosis by Peter Tsvetkov et al. [23]. Cuproptosis is closely associated with protein lipoylation which is concentrated in TCA cycle. The binding of copper to lipoylated components leads to lipoylated protein aggregation and subsequent iron-sulfur cluster protein loss, which causes proteotoxic stress and ultimately cell death [23]. Taken together, dysregulation of Cu homeostasis is generally observed in cancer and could serve as a potential target of anti-cancer therapy by inducing cuproptosis.

Liver plays a central role in regulating the systemic copper homeostasis. After being absorbed from the small intestine and delivered to the liver via portal circulation, copper ion can be released into the blood, transported into the bile for excretion or stored in hepatocytes [17, 24]. Except for its aforementioned functions under physiological processes, copper also involves in the tumorigenesis and development of HCC. An early study indicated that copper contents correlated with liver cirrhosis and HCC, and serum Cu levels might be helpful in detecting HCC [25]. Besides, high level of serum copper was associated with shorter survival of HCC patients [19, 26]. Caroline I Davis et al. observed that expression of Cu transporter genes, such as ATP7A, ATP7B, and SLC31A1, was significantly altered in tumor samples, which might be a cause for elevated Cu levels in the disease [27]. In recent years, position emission tomography (PET) using radioactive copper as a tracer has been explored for the application in HCC, relying on the altered copper metabolism in this tumor [28].

However, our understanding about the impact of abnormal copper metabolism on the development, treatment sensitivity and prognosis of HCC patients is still insufficient. Besides, genes regulating copper metabolism might also involve in cuproptosis and function as targets of anti-tumor therapy. Moreover, in the era of immunotherapy, how the alteration of copper metabolism modulates tumor microenvironment (TME) and the sensitivity to immune checkpoint inhibitors (ICIs) becomes an interesting question. In this work, we evaluated the alteration of copper metabolism-related pathways in HCC and established a copper metabolism- related risk score (CMscore). The CMscore was helpful in identifying HCC subtypes with distinct prognosis, pathway enrichment features and immune infiltrating signatures. In addition, the impact of CMscore on the sensitivity to transcatheter arterial chemoembolization (TACE) and ICIs was also investigated. Lastingly, WGCNA and subsequently correlation analysis revealed that SLC27A5 might regulate cuproptosis via FDX1 in HCC.

## MATERIALS AND METHODS

### Public data acquisition and processing

The data of the liver hepatocellular carcinoma project from the Cancer Genome Atlas (TCGA-LIHC) and from the International Cancer Genome Consortium (ICGC) database was downloaded as reported in our previous studies [29, 30]. The series matrix files and corresponding platform information of GSE14520, GSE25097, and GSE64041 were downloaded from Gene Expression Omnibus (GEO) database by the GEOquery package of R software. Data cleaning and processing was conducted as reported in our previous study [30]. The clinical information and gene expression data of the imvigor210 cohort were downloaded from the online website (http://research-pub.gene.com/IMvigor210CoreBiologies/) and was processed according to the instruction from the website. The data of patients with metastatic melanoma receiving immune-checkpoint inhibitor (ICI) was downloaded from the Supplementary Materials section of the work by David Liu et al. [31]. Since the data used in this study are all open data in public databases, an extra ethical approval was not required.

### Construction of copper metabolism-related risk score (CMscore)

The information of 14 copper metabolism-related pathways was downloaded from the Gene Set Enrichment Analysis (GSEA) website (https://www.gsea-msigdb.org/gsea, Supplementary Table 1). A total of 134 copper metabolism-related genes were identified (Supplementary Table 2. Univariate Cox regression analyses of these genes in both TCGA-LIHC and GSE14520 datasets were conducted and those genes with a prognostic significance less than 0.1 in both datasets were then input into a Least absolute shrinkage and selection operator (LASSO) regression model in the GSE14520 dataset, as reported in other studies [32, 33]. The parameters of the glmnet package to conduct LASSO regression analysis were set as follows: family = ‘cox’, nfolds = 10, type.measure = ‘deviance’. The generated crucial genes further underwent multivariate Cox regression analysis, which generated a coefficient of each crucial gene. A score of each sample was generated by multiplying the transcriptional value of each gene and its corresponding coefficient, and the calculation method was described below: score = -0.1984 * SORD - 0.1939 * TMPRSS6 - 0.1341 * CCS + 0.8732 * P2RX4 + 0.5949 * ATP13A2 + 0.6326 * LOX. The copper metabolism-related risk score (CMscore) was calculated with the formula reported in our previous study, namely, CMscore = (score-Min) / absolute (Max) [29].

### Enrichment analysis

The molecular function analysis of 134 copper related genes was conducted via the ClueGo application in the Cytoscape software [34]. Gene set variation analysis (GSVA) of the 14 copper metabolism-related pathways in HCC and corresponding normal liver samples was performed by the ‘GSEABase’ and ‘GSVA’ packages in R software [35]. GSEA of CMscore-based classification of HCC patients was performed as described in our previous works [36].

### Immune profile analysis

The immune score, stromal score and tumor purity of patients from TCGA-LIHC dataset was calculated by the ‘estimate’ package in R software [37]. The infiltration ratio of 22 types of immune cells in tumor microenvironment (TME) was calculated by CIBERSORT algorithm in R software [37]. The TIMER2.0 website (http://timer.cistrome.org/) also provided online evaluation of the fraction of 6 types of immune cells and the analyses were conducted in accordance with the instruction on the website [38].

### Weighted gene co-expression network analysis (WGCNA)

WGCNA was conducted in R software as reported in our previous study [33, 39]. Briefly, the transcriptional expression data of each dataset were used to construct a gene co-expression network after removing genes and samples with too many missing values. The correlation strength between the nodes was calculated by constructing an adjacency matrix based on the following formula:


$$S_{ij}=\left|cor\;\left(x_{i},x_{j}\right)\right|a_{ij}=S_{ij}^{\beta }$$

S_ij_ means the co-expression similarity and represents the Pearson’s correlation coefficient between two different genes i and j. X_i_ and X_j_ are the corresponding transcriptional expression values of the genes i and j, and α_ij_ is the correlation strength between the two genes. The scale-free R^2^ was set as 0.9 to select the corresponding soft-threshold β. One-step network construction and module detection methods were subsequently used, with a relatively large minimum module size of 200 and mergeCutHeight setting as 0.25 for the merging of modules. Finally, module–trait associations were quantified to identify modules significantly associated with CMscore. Besides, the definition and expression of module eigengenes (MEs), the gene significance (GS), and the module significance (MS) were similar with what had been described in a previous study [40].

### Immunohistochemistry (IHC) staining

Paraffin-embedded primary liver cancer tissues and the corresponding adjacent nontumorous samples (n =46) were obtained from the Pathology Department of Shanxi Provincial People’s Hospital. Patients’ information was exhibited in Supplementary Table 1. The HCC samples and corresponding tumor adjacent normal tissues were fabricated into a tissue chip. IHC staining of FFPE sections was performed as described in our previous studies [41, 42]. IHC samples were scored by two independent pathologists, according to criteria used in previous published studies [43, 44].

### Cell culture and treatment

Human liver cancer HepG2 and LM-3 cells were obtained from Cell Bank of Shanghai Institute for Biological Sciences, Chinese Academy of Sciences. Cells were cultured in DMEM medium, containing 10% FBS, and maintained in an incubator with constant temperature and CO2. Transfection was performed using EZ Trans Lipo (AC04L071, Life-iLab). The plasmids were acquired from Tianrun Aoke Biotechnology Co., Ltd (Yangling, China). The use of live cancer cells was approved by the Ethics Committee of Shanxi Provincial People's Hospital (2021-196).

### CCK-8 assay

In order to detect the effect of overexpression of SLC27A5 on the proliferation ability of liver cancer cells, HepG2 and LM-3 cells were seed into the 96-well plates, and treated with CCK-8 reagent (AC11L054, Life-iLab) at 0, 24, 48, and 72 hours, respectively. To detect the sensitivity of liver cancer cells to cuproptosis after overexpression of SLC27A5, HepG2 and LM-3 cells were in the 96 well plates, and treated with CCK-8 reagent 48 hours later. The absorbance value at a wavelength of 450 nm was measured using an enzyme labeling instrument.

### Western blotting

HepG2 and LM-3 cells were seeded into 6-well plates and conduct normal culture. Cells are collected when their density reaches 80%. RIPA lysate was used to lyse cells and obtain supernatant by centrifugation. The BCA kit (P0010, Beyotime) was used to determine the protein concentration. Western blotting was performed using antibodies against FDX1 (M05441, Boster), SLC27A5 (A09287-2, Boster), GAPDH (BM3876, Boster). Horseradish peroxidase-labeled Goat anti-rabbit IgG (H+L) (BA1039, Boster) was used as secondary antibodies.

### qRT-PCR

HepG2 and LM-3 cells were seeded into 6-well plates and conduct normal culture. Cells are collected when their density reaches 80%. TRIzol (AN51L758, Life-iLab) was used to lyse cells to obtain total RNA. BeyoRT™ II cDNA First Strand Synthesis Kit (D7168M, Beyotime) was used to obtained cDNA. 2x qPCR Mix (AN19L918, Life-iLab) was used to perform qPCR. The primers of target genes are as follows:

FDX1 forward, 5’-GTTCAACCTGTCACCTCATCTT-3’;

FDX1 Reverse 5’-CCAACCGTGATCTGTCTGTTAG-3’;

SLC27A5 forward, 5’-AGAGGACCGGACACATACA-3’;

SLC27A5 Reverse 5’-GTAGACTTCCCAGATCCGAATAG-3’;

GAPDH forward, 5’-GTCAAGGCTGAGAACGGGAA-3’;

GAPDH Reverse 5’-AAATGAGCCCCAGCCTTCTC-3’.

### Wound healing assay

HepG2 and LM-3 cells were seeded into 6-well plates and conduct normal culture. When the cell density reaches 80%, a 200 μL sterile pipette tip was used to scratch the cell layer and form a wound. The closure of the gap was imaged at designated time intervals using a microscope.

### Cell cycle assay

HepG2 and LM-3 cells were seeded into 6-well plates and subjected to starvation treatment for 12 hours, followed by normal cultivation for 24 hours. Cells were treated using a cell cycle assay kit plus (AC12L553, Life-iLab) and detected using flow cytometry. The difference between groups was detected by t-test.

### EdU staining

HepG2 and LM-3 cells were seeded into a special dish for confocal laser scanning microscopy. Cells were subjected to starvation treatment using a culture medium containing 1% FBS for 24 hours, followed by a complete culture medium and continued cultivation for 24 hours. BeyoClick™ EdU-555 Cell Proliferation Detection Kit (Beyotime, C0075L) was used for labeling Proliferated Cells, and DAPI was used to label the nucleus, then detected by confocal laser scanning microscopy.

### Statistical analysis

The data analyses and visualization were conducted in R (version 4.1.1), by using the following packages: ‘tidyverse’, ‘GEOquery’, ‘ggplot2’, ‘ggplotify’, ‘plot3D’, ‘dplyr’, ‘plyr’, ‘maftools’, ‘limma’, ‘survival’, ‘survminer’, ‘survivalROC’, ‘timeROC’, ‘cowplot’, ‘clusterProfiler’, ‘Hmisc’, ‘gridExtra’, ‘GSVA’, ‘corrplot’, ‘gmodels’, ‘VennDiagram’, and ‘pheatmap’. Wilcoxon test was used for comparison between two groups, whereas ANOVA test was for comparison among three groups. The Kaplan-Meier method was manipulated for prognosis analysis. Based on the optimal cutoff value of a marker determined by the ‘survminer’ package in R, HCC patients were divided into two subgroups [32, 45]. A *p*-value less than 0.05 was considered statistically significant (*, p < 0.05; **, p < 0.01; ***, p < 0.001; ****, p < 0.0001).

## RESULTS

### Distinct features of HCC in copper metabolism-related pathways

**Figure 1:**
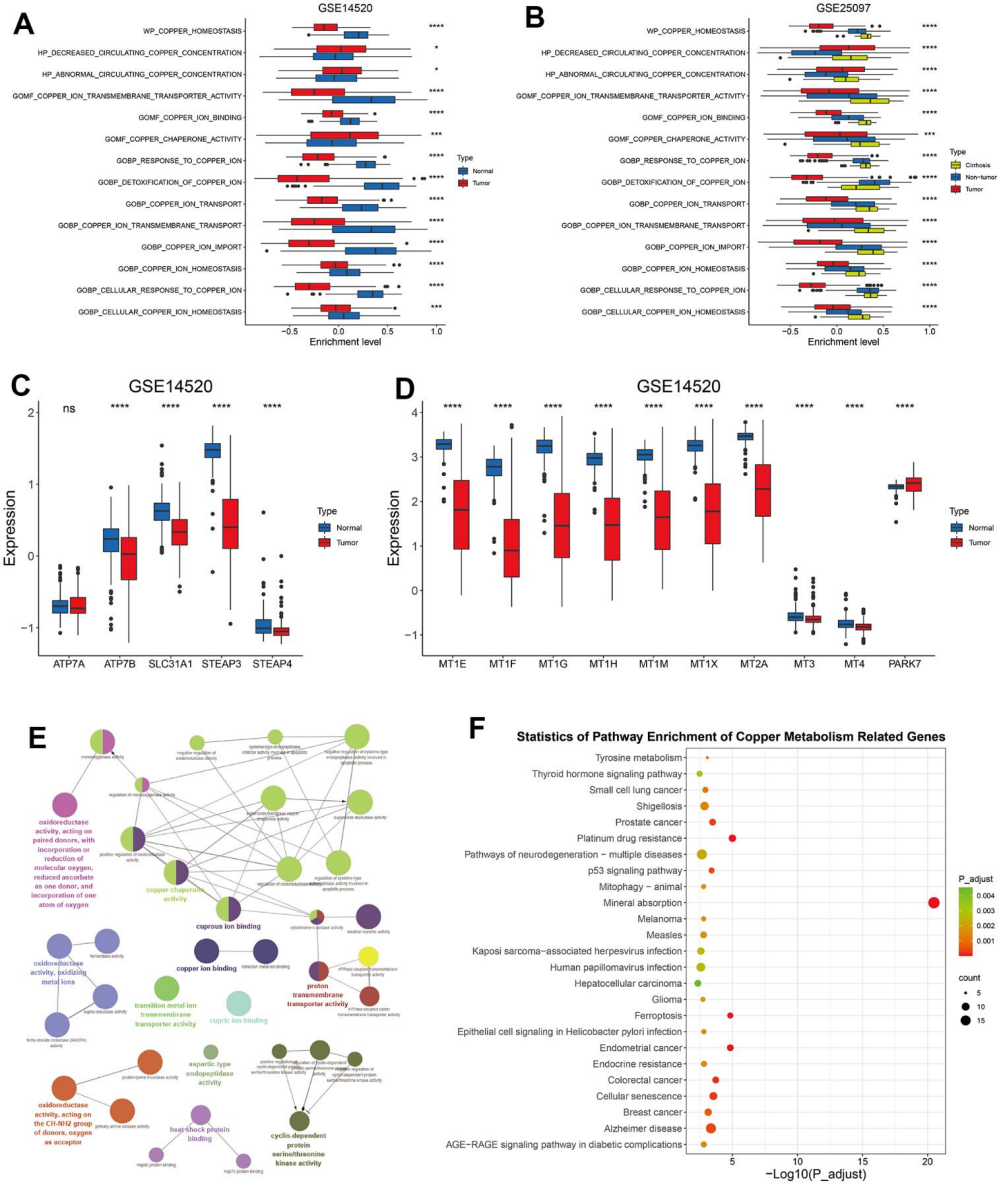
**The alteration of copper metabolism in HCC.** (**A**) The box plot showing the enrichment level of 14 copper metabolism in tumor and corresponding normal tissues from GSE14520 dataset. (**B**) The box plot showing the enrichment level of 14 copper metabolism in tumor, cirrhotic and normal livers from GSE25097 dataset. (**C**, **D**) The box plot showing the expression of copper ion import (**C**) or detoxification of copper ion (**D**) related genes in tumor and normal livers from the GSE14520 datasets. (**E**) The molecular function analysis of 134 copper metabolism-related genes in the GlueGo plug-in of the Cytoscape software. (**F**) KEGG analysis of 134 copper metabolism-related genes. P-values were shown as *p < 0.05, **p < 0.01, ***p < 0.001, and ****p < 0.0001.

Previous studies suggested that copper is tightly associated with liver cirrhosis and development of HCC, and with the survival of HCC patients [19, 25, 46]. To investigate the role of copper metabolism in HCC, we searched GSEA website online with the keyword “copper” and identified 14 copper metabolism-related pathways containing 134 unique genes (Supplementary Tables 2, 3). The GSVA enrichment analyses of these 14 pathways in HCC and corresponding normal liver samples revealed that HCC samples exhibited decreased level of most copper metabolism-related pathways, like “GOMF_COPPER_ION_TRANSMEMBRANE_TRANSPORTER_ACTIVITY”, “GOBP_RESPONSE_TO_COPPER_ION”, “GOBP_DETOXIFICATION_OF_COPPER_ION”, and “GOBP_COPPER_ION_IMPORT” (Figure 1A and supplementary Figure 1A, 1B). Correspondingly, most copper import related genes (Figure 1C) and detoxification of copper ion related genes (Figure 1D) were significantly downregulated in HCC, when compared with normal liver tissues. A previously study indicated that liver tissues with cirrhosis showed an accumulation of copper when compared with healthy ones [47]. We noticed that liver tissues with cirrhosis had a further increased level than HCC and normal tissues in some terms like “GOMF_COPPER_ION_TRANSMEMBRANE_TRANSPORTER_ACTIVITY”, “GOBP_COPPER_ION_TRANSMEMBRANE_TRANSPORT”, and “GOBP_COPPER_ION_TRANSPORT” (Figure 1B), probably reflecting a response of cirrhotic tissues to the elevated level of copper ions. Taken together, these results indicated that HCC might show resistance to copper ion influx and decreased capability of detoxification of copper ion.

Copper ion plays a critical role in mitochondrial respiration, antioxidant defense, biosynthesis of neurotransmitters and other biological activities [48]. In addition to copper ion binding and transporting, these 134 copper metabolism-related genes were also found to be enriched in molecular functions like “oxidoreductase activity”, “heat shock protein binding”, and “cyclin dependent protein serine/threonine kinase activity” (Figure 1E). KEGG analyses revealed that these genes involved in mineral absorption and in other terms like prostate cancer, colorectal cancer, platinum drug resistance, and ferroptosis (Figure 1F).

### Construction and validation of copper metabolism-related risk score (CMscore)

**Figure 2:**
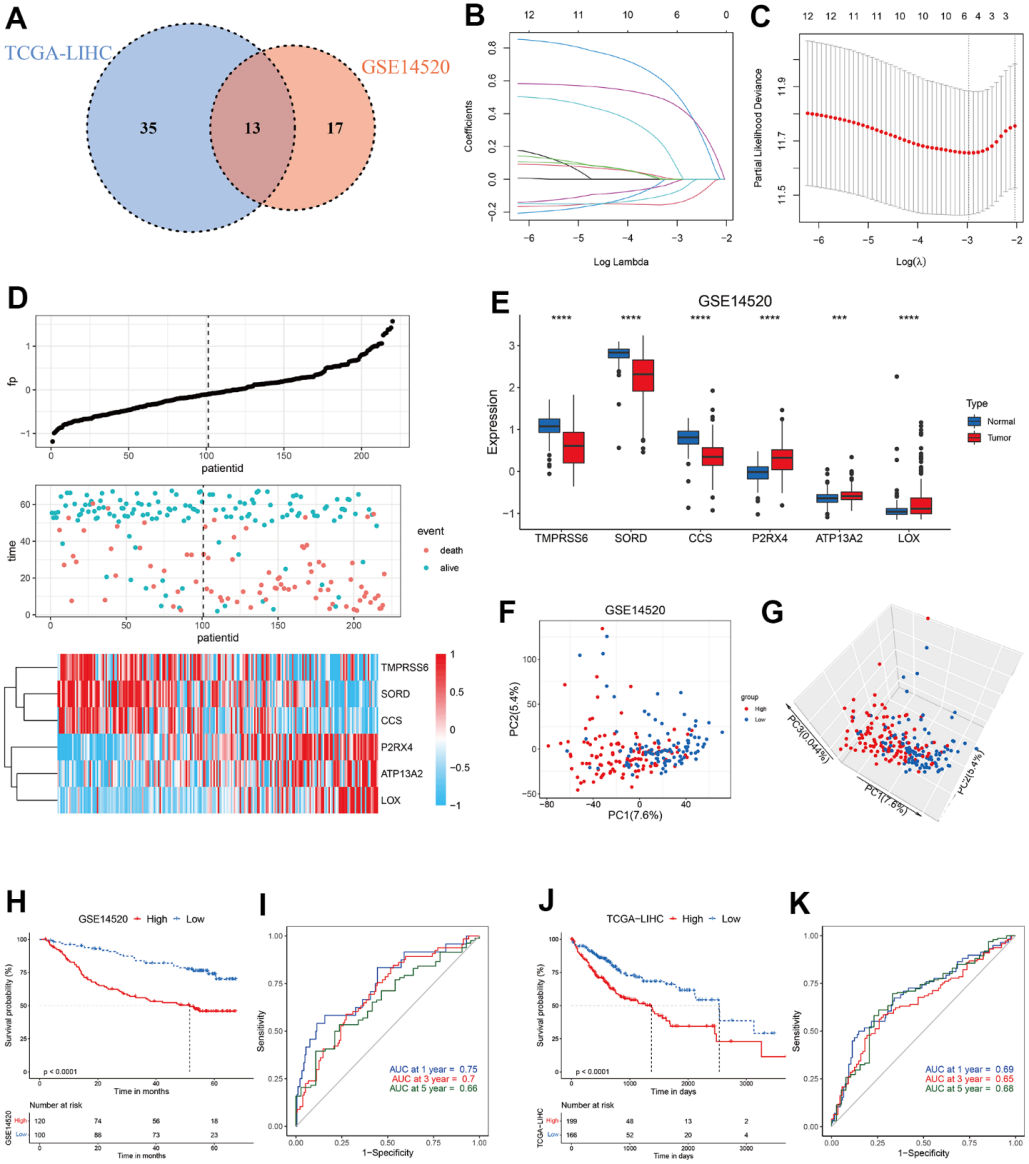
**Construction of CMscore.** (**A**) Venn diagram indicating 13 copper metabolism-related genes with prognostic significance less than 0.1 in GSE14520 and TCGA-LIHC datasets. (**B**, **C**) The LASSO Cox regression model was constructed from 13 copper metabolism-related genes. The tuning parameter (λ) was calculated based on the partial likelihood deviance with ten-fold cross validation. An optimal log λ value shown by the vertical black dot-lines in the plots. The six signature genes were identified according to the best fit profile. (**D**) The distribution and optimal cutoff value of CMscore, the OS status of each sample, and the expression value of the six crucial genes in the GSE14520 dataset. (**E**) The box plot showing the expression of six crucial genes in tumor and normal livers from the GSE14520 datasets. (**F**, **G**) The 2D (**F**) and 3D (**G**) plots of the PCA of the GSE14520 dataset based on the expression profiles of the 6 signature genes. (**H**) The prognostic significance of CMscore in GSE14520. The Kaplan-Meier method was used for prognosis analysis. (**I**) Time-dependent ROC analyses of the CMscore regarding the OS and survival status in the GSE14520 dataset. (**J**) The prognostic significance of CMscore in TCGA-LIHC. The Kaplan-Meier method was used for prognosis analysis. (**K**) Time-dependent ROC analyses of the CMscore regarding the OS and survival status in the TCGA-LIHC dataset. P-values were shown as *p < 0.05, **p < 0.01, ***p < 0.001, and ****p < 0.0001.

Since copper concentration shows correlation with survival of HCC patients and high level of copper in cytoplasm could induce cuproptosis [19, 23], we hypothesized that copper metabolism-related genes might help to identify specific subtypes with distinct prognosis and sensitivity to copper-dependent death. We first conducted univariate Cox regression analyses of these 134 copper metabolism-related genes in TCGA-LIHC and GSE14520 datasets, and found 13 genes showed prognostic significance with *p*-value less than 0.1 in both datasets (Figure 2A). The 13 genes were then input into a LASSO regression model in the GSE14520 dataset and 6 crucial genes were obtained (Figure 2B, 2C). A novel risk score, which was named CMscore were calculated by the method described in the Method and Materials Section. As shown in Figure 2D, patients with high CMscore had lower expression of TMPRSS6, SORD and CCS, whereas had higher expression of P2RX4, ATP13A2 and LOX. In addition, TMPRSS6, SORD and CCS all showed significantly decreased expression in HCC (Figure 2E) and their high expression was correlated with longer survival time (Supplementary Figure 1C–1E). On the contrary, P2RX4, ATP13A2 and LOX showed significantly upregulated level in HCC (Figure 2E) and their high expression was correlated with dramatically shorter survival time (Supplementary Figure 1F–1H). PCA analyses revealed that HCC patients with high- or low-CMscore were distinctly clustered (Figure 2F, 2G). In the GSE14520 datasets, HCC patients with low CMscore (100/220, 45.45%) had considerably longer median overall survival (OS) than those with high CMscore (not reached vs. 51.60 months, p < 0.0001, Figure 2H), and the AUC reached 0.75 at 1 year, 0.7 at 3 years, and 0.66 at 5 years (Figure 2I). Likely, when 45.45% of HCC patients in the TCGA-LIHC and ICGC-LIRI datasets were classified into the low-CMscore subgroup according to the order of CMscore, these patients also exhibited significantly longer OS (p < 0.0001 or = 0.008, respectively, Figure 2J and Supplementary Figure 2A). The AUC of 1-year, 3-year and 5-year reached 0.69, 0.65, and 0.68 in the TCGA-LIHC, respectively (Figure 2K). And that of 1-year and 3-year was 0.76 and 0.67 in the ICGC-LIRI dataset (Supplementary Figure 2B). In addition, HCC patients with low CMscore also had significantly longer progression free survival (PFS, p = 0.0012 or = 0.028, respectively, Supplementary Figure 2C, 2D).

### CMscore serves as an independent risk score

**Figure 3:**
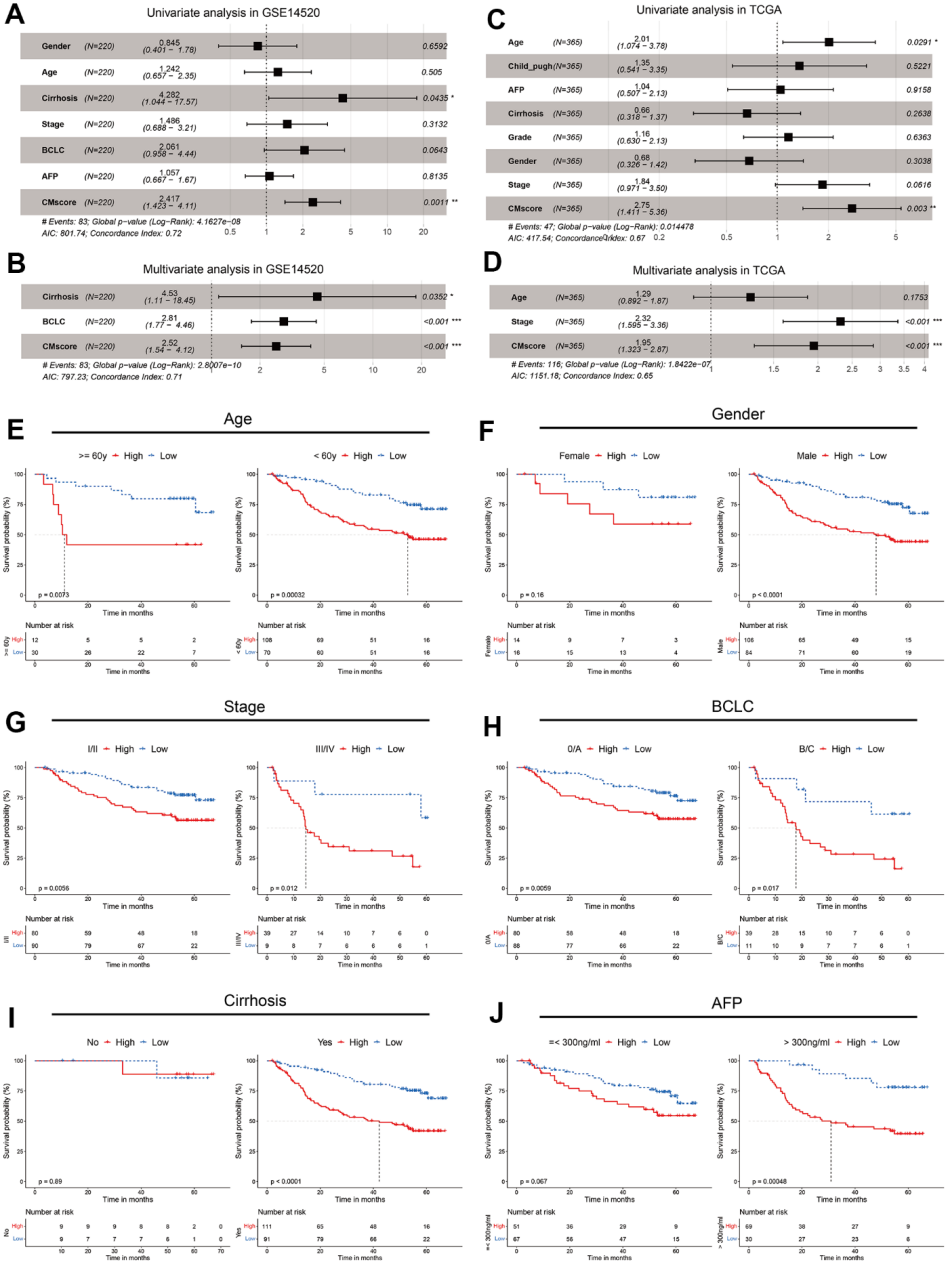
**CMscore was an independent prognostic predictor for HCC patients.** (**A**, **B**) Results of the univariate (**A**) and multivariate (**B**) Cox regression analyses regarding OS in the GSE14520 cohort. (**C**, **D**) Results of the univariate (**C**) and multivariate (**D**) Cox regression analyses regarding OS in the TCGA-LIHC cohort. (**E**–**J**) Prognostic analyses of CMscore in subgroups of HCC patients stratified by age (**E**), gender (**F**), stage (**G**), BCLC stage (**H**), cirrhosis status (**I**) and AFP level (**J**).

To further evaluate the impact of CMscore on prognosis of HCC patients, we conducted univariate Cox analyses and subsequently multi-variate Cox analyses in GSE14520 and TCGA-LIHC, including CMscore and available clinical factors (Figure 3A–3D). The results showed that CMscore was an independent prognostic factor in both datasets, after correction for other confounding clinical features (p < 0.001, Figure 3B, 3D). Indeed, except female patients, HCC patients with high CMscore had significantly shorter OS in subgroup prognostic analyses (Figure 3E, 3H), supporting an independent role of CMscore in the survival of HCC patients.

We also analyzed the associations between CMscore and clinicopathological features of these patients. As shown in Table 1, although a higher percentage of HCC patients in the low CMscore subgroup were >= 60 years in the GSE14520 dataset, such a significant association was not observed in the TCGA-LIHC dataset (p = 0.528). A similar inconsistence was also observed between CMscore and gender (Table 1). Besides, CMscore showed no association with the fibrosis or cirrhosis state of HCC patients (p = 0.806 or > 0.999). However, a significantly higher percentage of HCC patients with high CMscore had AFP level great than 300 ng/ml (p < 0.001), were at advanced stage (III/IV or BCLC B/C stage, Table 1), and had worse histologic grade (G3 or G4, p = 0.002, Table 1).

**Table 1:** Relationships between CMscore and clinicopathological features of HCC patients.

		**GSE14520**			**TCGA**		
		**Low CMscore**	**High CMscore**	**p-value**	**Low CMscore**	**High CMscore**	**p-value**
Age	<60	70	108	< 0.001	72	93	0.528
	≥60	30	12		94	106	
Gender	male	84	106	0.431	125	121	0.004
	female	16	14		41	78	
Fibrosis/Cirrhosis	No	9	9	0.806	39	35	> 0.999
	Yes	91	111		72	63	
AFP(ng/ml)	≤300	67	51	< 0.001	118	94	< 0.001
	>300	30	69		20	44	
Stage	I/II	90	80	< 0.001	126	128	0.018
	III/IV	9	39		30	57	
BCLC	0/A	88	80	< 0.001			
	B/C	11	39				
Child_pugh	A				118	65	> 0.999
	B/C				12	7	
Histologic_grade	G1+G2				119	111	0.002
	G3+G4				45	85	

### Pathway enrichment analyses

**Figure 4:**
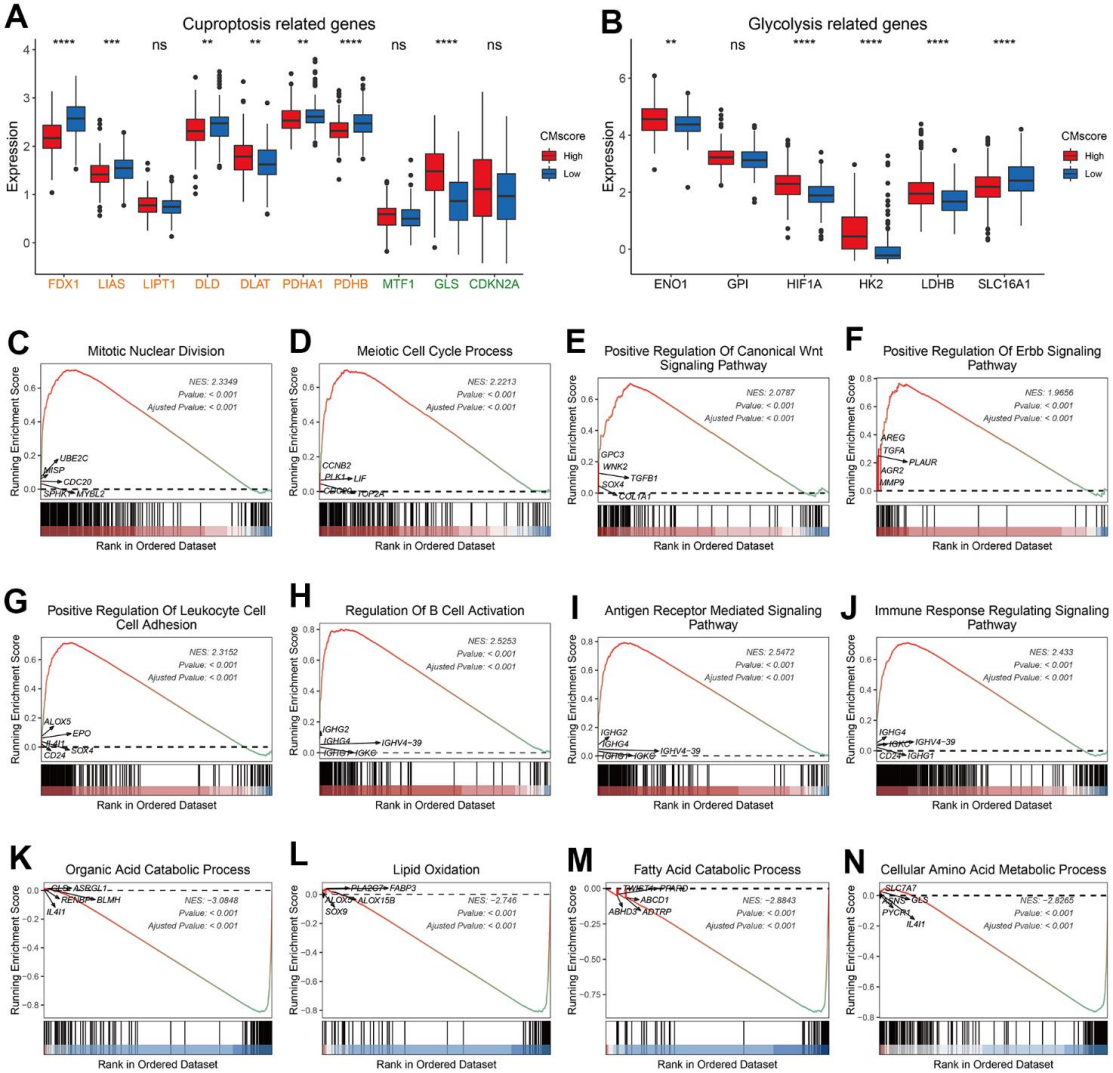
**Pathway enrichment analyses of CMscore-based HCC groups.** (**A**, **B**) The box plot showing the expression of cuproptosis (**A**) and glycolysis (**B**) related genes in high- and low-CMscore subgroups of the TCGA_LIHC dataset. (**C**–**N**) GSEA of the high- (**D**) and low-CMscore (**E**) subgroup in the TCGA-LIHC cohort. P-values were shown as *p < 0.05, **p < 0.01, ***p < 0.001, and ****p < 0.0001.

Cuproptosis is recently identified copper-independent cell death in which excess copper promotes the aggregation of lipoylated proteins and subsequent proteotoxic stress [23]. Currently, 10 genes have been showed to regulate the sensitivity of cuproptosis. Among these genes, FDX1, LIAS, LIPT1, DLD, DLAT, PDHA1, and PDHB are found to facilitate cuproptosis, whereas MTF1, GLS and CKDN2A inhibit the cell death [23]. As shown in Figure 4A, most pro-cuproptosis genes, like FDX1, LIAS and PDHB, were significantly downregulated in HCC patients with high CMscore, whereas GLS, an anti-cuproptosis gene, was significantly upregulated in these patients. Peter Tsvetkov et al. also found that cells more reliant on mitochondrial respiration show a dramatically increase in sensitivity to copper ionophores than those undergoing glycolysis and growing cells in hypoxic conditions led to attenuation of cuproptosis [23]. We noticed that most glycolysis related genes showed a significantly higher expression in HCC patients with high CMscore (Figure 4B). Taken together, these results indicated that HCC patients might be more resistant to cuproptosis.

GSEA analyses revealed that HCC patients with high CMscore were enriched in cancer-related pathways like mitotic nuclear division, meiotic cell cycle process, WNT signaling pathway, and ERBB signaling pathway (Figure 4C–4F and Supplementary Table 4), and in immune related pathways like positive regulation of leukocyte cell cell adhesion (Figure 4G–4J, Supplementary Table 4), whereas patients with low CMscore were enriched in metabolism-related pathways like organic acid catabolic process and lipid oxidation (Figure 4K–4N and Supplementary Table 5).

### Immune landscape of CMscore stratified HCC patients

**Figure 5:**
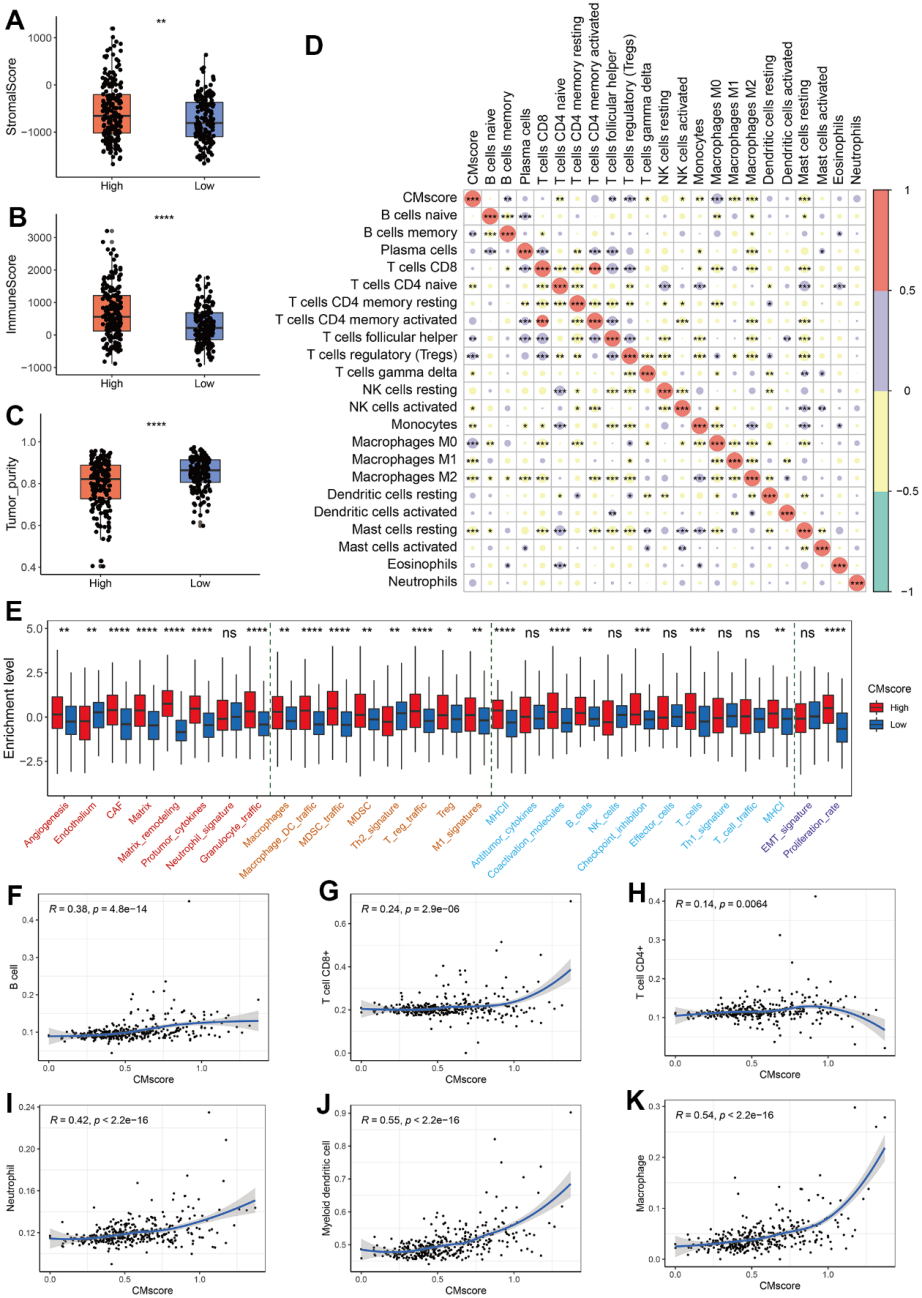
**Immune profile of CMscore-based HCC groups.** (**A**–**C**) Box and dot plot showing stromal score (**A**), immune score (**B**), and tumor purity of HCC patients from high- and low-CMscore subgroups. Wilcoxon test was used for data analyses. (**D**) Correlation analyses between CMscore and infiltration of 22 types of immune cells estimated by CIBERSORT method. (**E**) The box plot showing the enrichment score of 29 Fregs in HCC patients from the low- or high-CMscore subgroups. The “Angiogenesis Fibroblasts” related terms were marked with red font, the “Pro-tumor immune infiltrate” related terms were with yellow font, the “Anti-tumor immune infiltrate” related terms were with blue font, and “EMT signature / proliferation rate” related terms were with purple font. (**F**–**K**) Correlation analyses between CMscore and infiltration of 6 types of immune cells estimated by the TIMER website online. P-values were shown as *p < 0.05, **p < 0.01, ***p < 0.001, and ****p < 0.0001.

To better character the immune landscape of HCC patients stratified by CMscore, we first calculate the immune score, stromal score and tumor purity of each sample in the TCGA-LIHC dataset. As shown in Figure 5A–5C, HCC patients with high CMscore had significantly higher immune score and stromal score, and correspondingly lower tumor purity. A significantly positive correlation was observed between CMscore and B cell memory, T cell follicular helper, T cells regulatory, or M0 macrophages (Figure 5D). On the contrary, CMscore showed a significantly negative correlation with T cells CD4 naïve, T cells gamma delta, NK cells activated, monocytes, M1 or M2 macrophages, and mast cells resting (Figure 5D).

Alexander Bagaev et al. clustered TME properties via a list of 29 functional gene expression signatures (Fges) [49]. We found that HCC patients with high CMscore had elevated expression of most Fregs associated with angiogenesis fibroblasts (CAFs), such as angiogenesis, matrix remodeling, and protumor cytokines. Besides, the expression of all pro-tumor immune infiltrate associated Fges and part of anti-tumor immune infiltrate associated Fges (MHCI, MHCII, coactivation molecules, B cells, checkpoint inhibition, and T cells) were significantly elevated in HCC patients with high CMscore a higher proliferation rate (Figure 5D). In addition, high-CMscore HCC patients also showed an increase in proliferation rate (Figure 5D), which is consistent with the GSEA results (Figure 4C, 4D).

Tumor Immune Estimation Resource (TIMER; cistrome.shinyapps.io/timer) helps to calculate the levels of six tumor-infiltrating immune subsets, namely B cell, CD4+ T cell, CD8+ T cell, neutrophil, myeloid dendritic cell and macrophage [50]. We also found that CMscore exhibited a positive association with the infiltration of all these cells (Figure 5F–5K). In particular, CMscore had a strong correlation with the fraction of myeloid dendritic cell or macrophage (R = 0.55 or = 0.54, respectively, Figure 5J, 5K).

### Association between CMscore and treatment of HCC

**Figure 6:**
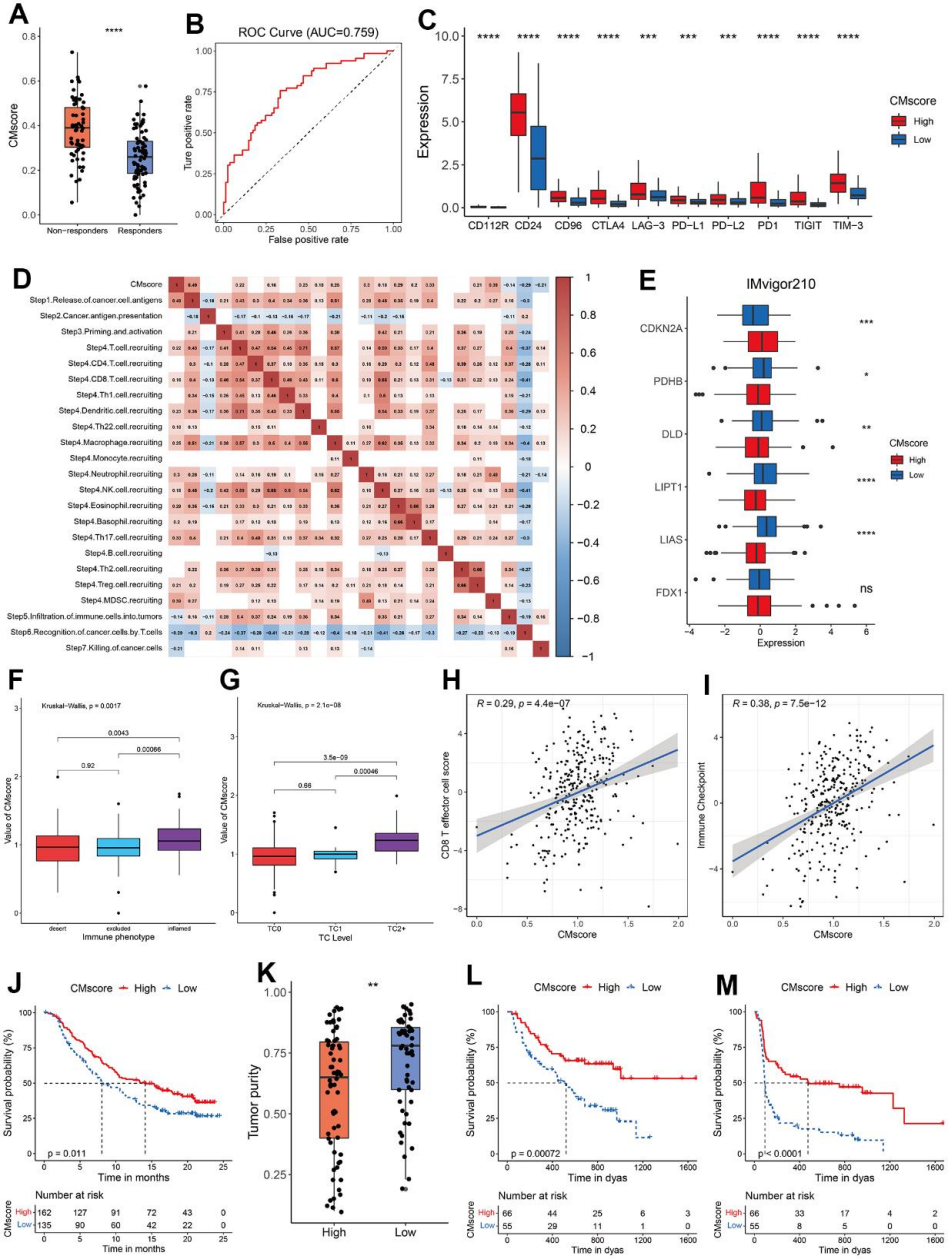
**Guidance of CMscore in the therapy for HCC patients.** (**A**) The box and dot plot showing the value of CMscore between responders and non-responders to TACE. Wilcoxon test was used for data analyses. (**B**) The AUC value of CMscore in predicting non-responsiveness of HCC patients to TACE. (**C**) The boxplot showing the expression of ICI-related genes in high- and low-CMscore subgroups of the TCGA_LIHC dataset. (**D**) Correlation between CMscore and immune activity scores of each step of the Cancer-Immunity Cycle. (**E**) The boxplot showing the expression of cuproptosis-related genes in high- and low-CMscore subgroups of the IMvigor210 cohort. (**F**) The boxplot showing the level of CMscore in three immune phenotypes of mUC patients in the IMvigor210 cohort. (**G**) The boxplot showing the level of CMscore in mUC patients with different TC level in the IMvigor210 cohort. (**H**) Correlation between CMscore and CD8 T effector cell score in the IMvigor210 cohort. (**I**) Correlation between CMscore and level of immune checkpoint in the IMvigor210 cohort. (**J**) OS analyses of mUC patients with high and low CMscore in the IMvigor210 cohort. (**K**) Box and dot plot showing the tumor purity of metastatic melanoma patients with high and low CMscore. (**L**, **M**) OS (**L**) and PFS (**M**) analyses of metastatic melanoma patients with high and low CMscore. P-values were shown as *p < 0.05, **p < 0.01, ***p < 0.001, and ****p < 0.0001.

TACE, a recommended treatment for HCC patients at intermediate stage, exerts anti-tumor function by injecting drugs into the artery supplying for HCC nodules [51]. We noticed that HCC patients responding to TACE had a significantly lower level of CMscore (p < 0.0001, Figure 6A), and the AUC of CMscore in predicting the non-responsiveness reached 0.759 (Figure 6B). As demonstrated in our previous section, HCC patients with high CMscore had increased proliferation rate (Figure 5E), and decreased expression of pro-cuproptosis related genes (Figure 4A). Similarly, HCC patients responding to TACE had significantly higher level of MKI67, a marker of cell proliferation, and of CDKN2A, an anti-cuproptosis gene (Supplementary Figure 2F), and these patients also had significantly decreased expression of FDX1, a key pro-cuproptosis gene (Supplementary Figure 2F).

Besides, immune checkpoint inhibitors (ICIs), like pembrolizumab, nivolumab and sintilimab, have become part of the first- or second-line therapy for advanced HCC patients [52, 53]. As shown in Figure 6C, HCC patients with high CMscore had a significantly higher expression of all major ICI targets, including PD-L1, CTLA4, CD24 and TIGIT. Previously, a series of stepwise events is depicted by Liwen Xu et al. during the anticancer immune response [54]. As shown in Figure 6D, CMscore had a significantly negative association with step 5 to step 7, whereas showed a significantly positive correlation with the release of cancer cell antigens (step 1), and recruiting of many types of immune cells (step 4). To further evaluate the impact of CMscore on tumor’s response to immunotherapy, we first investigated a large cohort of patients with metastatic urothelial cancer (mUC) treated with atezolizumab, an anti-PD-L1 agent (the IMvigor210 study) [55]. We calculated the CMscore of each sample in the cohort and 45.45% of these patients were classified into the low-CMscore subgroup based on the order of CMscore, as described in the aforementioned section. Similarly, mUC patients with high CMscore had significantly decreased level of most pro-cuproptosis related genes such as LIAS, LIPT1 and DLD, and upregulated level of CDKN2A, an anti-cuproptosis gene (Figure 6E). Besides, mUC patients from the inflamed cluster, or those with TC2+, had the highest level of CMscore (Figure 6F and Supplementary Figure 2G). CMscore also showed a positive correlation with CD8 T effector cell score and level of immune checkpoint (Figure 6H, 6I), and these results were consistent with those from the HCC cohort (Figures 5E, 5H, 6C). After treatment of atezolizumab, mUC patients from the high-CMscore subgroup exhibited significantly longer OS than those from the low-CMscore one (median OS, 14.13 months vs. 8.08 months, p = 0.011, Figure 6J). In another large cohort in which 144 patients with metastatic melanoma treated with anti-PD1 ICIs (121 of them with survival data available) [31], we also calculated the CMscore of each sample and categorized 45.45% of them into the low-CMscore subgroup. Again, we found that melanoma patients with high CMscore had significantly lower tumor purity (Figure 6K), consistent with the results from the HCC cohort (Figure 5C). Particularly, these melanoma patients with low CMscore exhibited dramatically shorter OS (p = 0.00072, Figure 6L) and PFS (p < 0.0001, Figure 6M).

### Identification of potential target of cuproptosis

**Figure 7:**
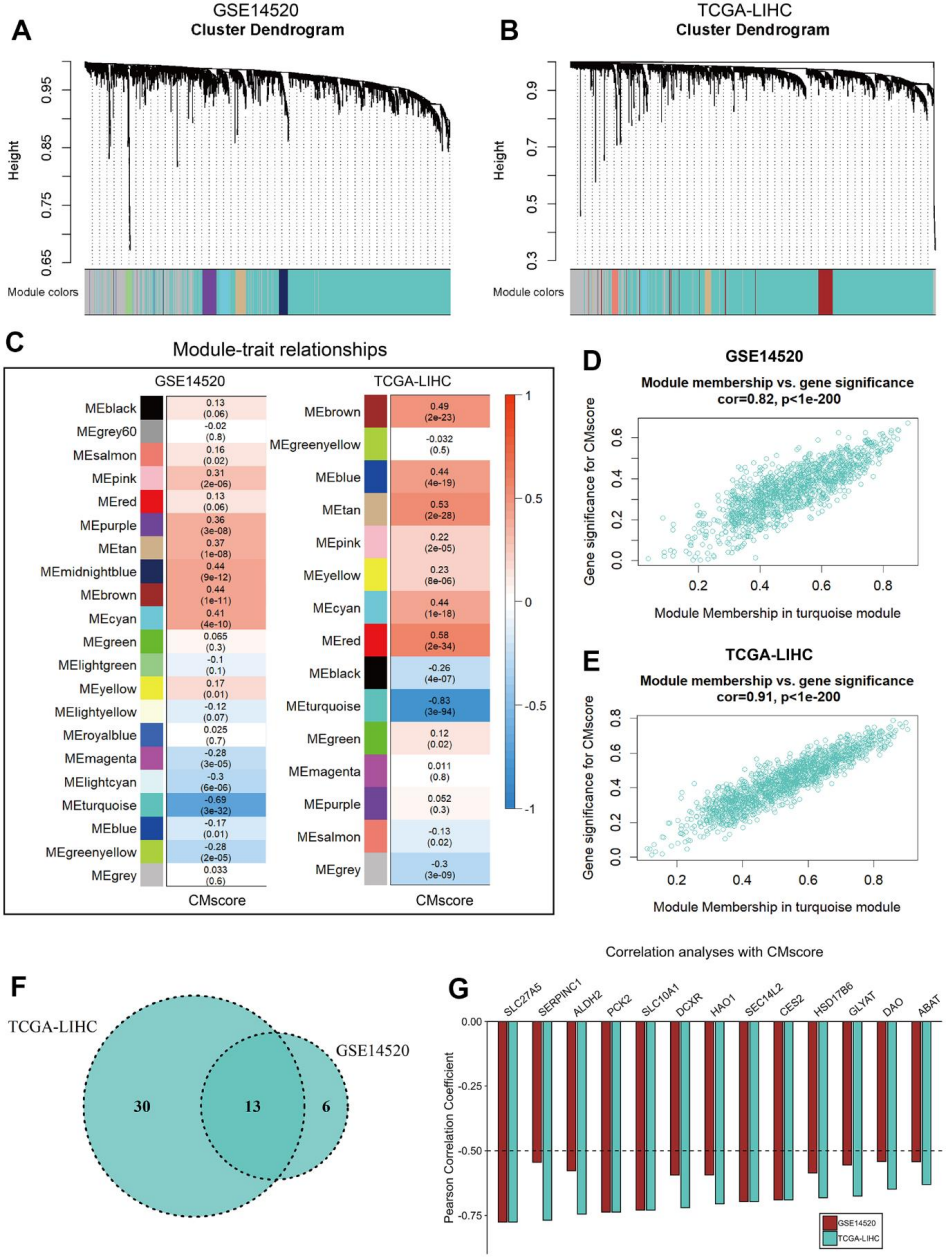
**WGCNA analyses of CMscore.** (**A**, **B**) Merging of mRNA co-expression modules in GSE14520 (**A**) and TCGA-LIHC (**B**) datasets. (**C**) Correlation heatmap of module genes and CMscore in the GSE14520 and TCGA-LIHC datasets. The correlation coefficient changed from –1 to 1 as the color turned from blue to red gradually. (**D**, **E**) Scatterplot of the correlation coefficient between the selected module (turquoise module) and the CMscore in GSE14520 (**D**) and TCGA-LIHC (**E**) datasets. (**F**) Venn plot of hub genes of the selected module in GSE14520 and TCGA-LIHC datasets. (**G**) Correlation coefficient of selected hub genes with CMscore in GSE14520 and TCGA-LIHC datasets.

To identify potential genes involving copper metabolism and cuproptosis, WGCNA was applied to identify modules highly correlated with CMscore in both GSE14520 and TCGA-LIHC datasets (Figure 7A, 7B). The turquoise modules in the GSE14520 and TCGA-LIHC datasets showed strongest association with CMscore (Figure 7C–7E). To filter the hub genes highly correlated with CMscore, we selected genes which had a gene significance value greater than 0.2 and module membership value greater than 0.8 for further analyses. As shown in Figure 7F, 13 hub genes both existed in the turquoise modules of TCGA-LIHC and GSE14520 datasets, and these genes all showed a strong correlation with CMscore (Figure 7G).

**Figure 8:**
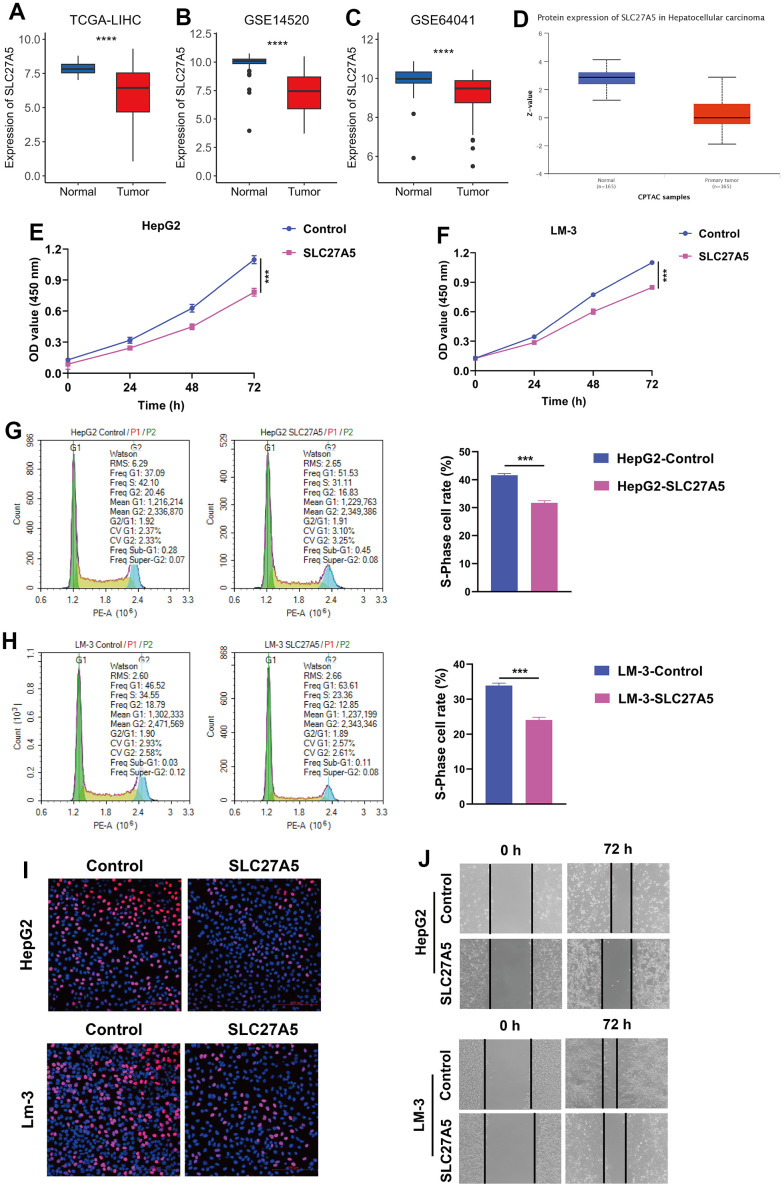
**The expression and function of SLC27A5 in HCC.** (**A**–**C**) The transcriptional expression of SLC27A5 in HCC and normal tissues of TCGA-LIHC (**A**), GSE14520 (**B**) and GSE64041 (**C**) datasets. (**D**) The protein level of SLC27A5 in HCC and normal tissues. (**E**, **F**) CCK-8 assay for HepG2 (**E**) and LM-3 (**F**) cells overexpressing SL27A5. (**G**, **H**) HepG2 (**G**) and LM-3 (**H**) cells were subject to flow cytometry analysis for cell cycle. (**I**) HCC cells overexpressing SLC27A5 were subject to EdU staining. (**J**) HCC cells overexpressing SLC27A5 were subject to wound healing assay.

**Figure 9:**
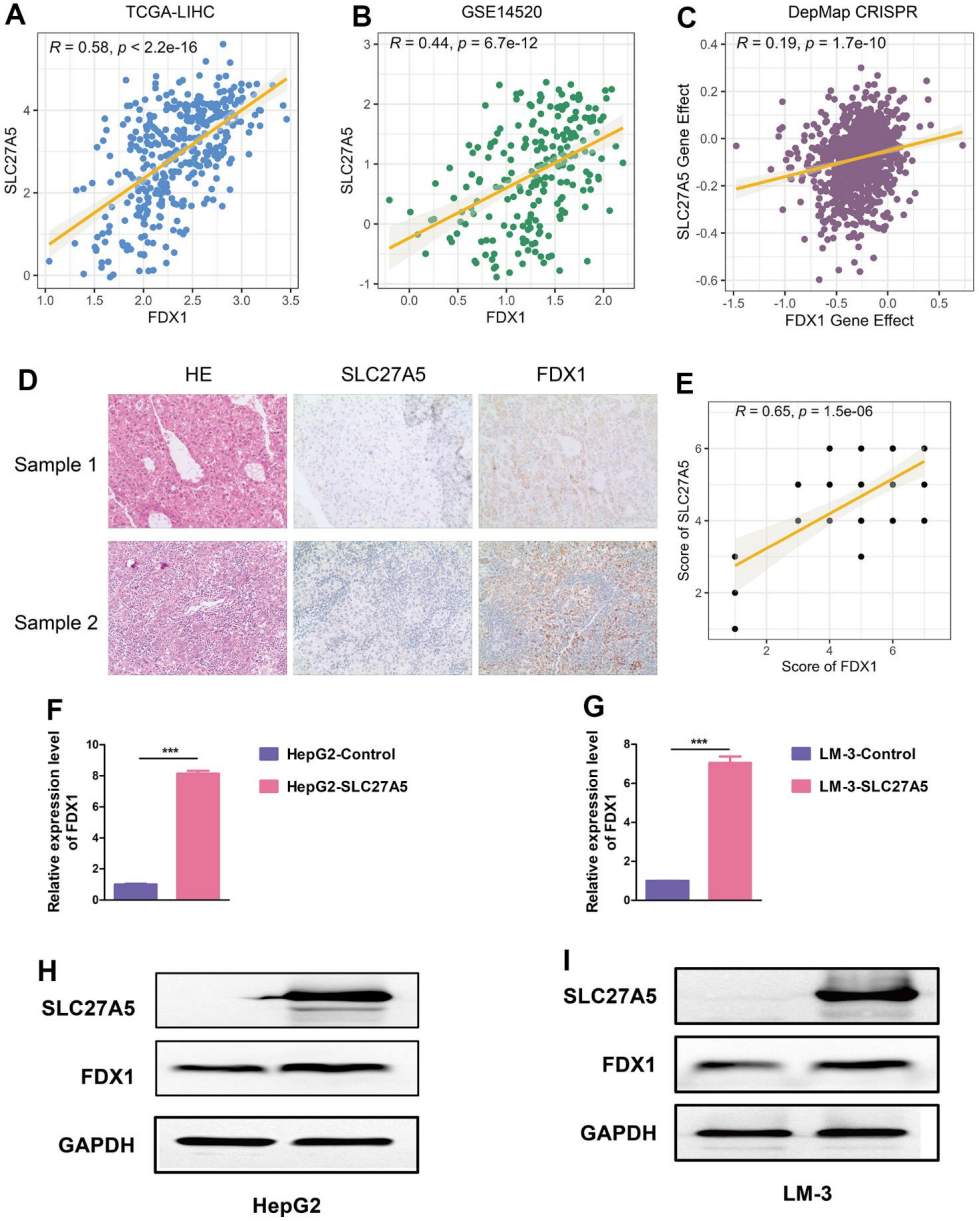
**SLC27A5 upregulates FDX1 in HCC.** (**A**, **B**) The correlation between the expression of SLC27A5 and that of FDX1 in TCGA-LIHC (**A**) and GSE14520 (**B**) datasets. (**C**) Dot plot showing the dependency scores for SLC27A5 and FDX1 across all tumor cell lines in the Project Achilles/Cancer Dependency Map Portal (DepMap). (**D**, **E**) The expression of and correlation between SLC27A5 and FDX1 in collected HCC tissue chip. (**F**, **G**) HepG2 (**F**) and LM-3 (**G**) cells overexpressing SLC27A5 were subject to qRT-PCR analysis. (**H**, **I**) Western blotting using lysates of HepG2 (**H**) and LM-3 (**I**) cells after overexpressing SLC27A5.

Since SLC27A5 showed the strongest negative correlation with CMscore (Figure 7G), we further evaluated its relationship with cuproptosis. As shown in Figure 8A–8D, the transcriptional and protein level of SLC27A5 were significantly downregulated in HCC when compared with normal tissues. To investigate the role of SLC27A5 in liver cancer, we overexpressed SLC27A5 in HepG2 and LM-3 cells. CCK-8 assay indicated that overexpression SLC27A5 caused decreased cell proliferation of HCC cells (Figure 8E, 8F). The results of flow cytometry suggested that the proportion of S phase HCC cells decreased after overexpression of SLC27A5 (Figure 8G, 8H). EdU staining results also showed a significant decrease in the proliferation rate of HCC cells after overexpression of SLC27A5 (Figure 8I). Besides, HCC cells with overexpression of SLC27A5 showed a slower migration rate than the control groups (Figure 8J). Considering FDX1 plays a central role in the induction of cuproptosis [23], we further investigated the relationship between SLC27A5 and FDX1. The transcriptional level of SLC27A5 exhibited a strong positive correlation with that of FDX1 in TCGA-LIHC (Figure 9A, r = 0.58, p < 2.2e-16) and in GSE14520 (Figure 9B, r = 0.44, p < 6.7e-12). Further, analysis of the Cancer Dependency Map Portal (DepMap) [56] revealed that SLC27A5 exhibited a significant co-dependency with FDX1 (Figure 9C, r = 0.19, p = 1.7e-10). IHC staining of SLC27A5 and FDX1 in a collected HCC tissue chip also confirmed a significantly positive correlation between the expression of SLC27A5 and that of FDX1 (Figure 9D, 9E, r = 0.65, p = 1.5e-06). Lastly, the qRT-PCR and western blotting experiments all confirmed that both transcriptional and protein level of FDX1 were significantly upregulated in HCC cells after overexpression SLC27A5 (Figure 9F–9I). These findings indicate that SL27A5 may promote cuproptosis by upregulating FDX1 in HCC.

## DISCUSSION

Copper ion is essential in various biological activities, including cellular respiration, intracellular signaling transduction, neuropeptide processing, cell proliferation and angiogenesis [9, 11]. Dysregulated copper metabolism has been observed in many types of cancer, and elevated copper level is associated with more aggressive features of tumor and worse prognosis of cancer patients [11, 13, 14, 26]. In this work, we noticed that features of copper metabolism in HCC are indeed distinct from normal or cirrhotic livers (Figure 1A–1D). GSVA analyses of 14 copper metabolism-related pathways showed that HCC samples had decreased expression of most terms, like “GOMF_COPPER_ION_TRANSMEMBRANE_TRANSPORTER_ACTIVITY”, “GOBP_RESPONSE_TO_COPPER_ION”, “GOBP_DETOXIFICATION_OF_COPPER_ION”, and “GOBP_COPPER_ION_IMPORT” (Figure 1A, 1B). The expression of four metal-binding metallothioneins (MTs): MT1, MT2, MT3, and MT4, was all dramatically decreased in HCC samples (Figure 1D). MTs can detect, store, and transport copper, and protect cells from copper toxicity by chelating excess copper ions [57]. The abnormal expression of MTs might contribute to the dysregulated copper metabolism in HCC cells and also render these cells more susceptible to copper-dependent cell death, namely cuproptosis [23].

To better characterize the change of copper metabolism in HCC, we incorporated the genes involved in copper metabolism-related pathways, and constructed the novel risk score, namely CMscore (Figure 2A–2D). The risk score was based on the expression of six crucial genes, and these genes were TMPRSS6, SORD, CCS, P2RX4, ATP13A2 and LOX. The first three genes might function as tumor-suppressors in HCC, since they expression was dramatically decreased in tumors when compared with normal livers (Figure 2E), and their low expression was associated with significantly shorter OS (Supplementary Figure 1C–1E). On the contrary, the latter three genes might act as oncogenes because their expression and relationship with prognosis was opposite to that of the first three genes (Figure 2E and Supplementary Figure 1F–1H). TMPRSS6 is mainly expressed in the liver and critical in maintaining iron homeostasis [58], and it also contributes to abnormal circulating copper concentration (Supplementary Table 1). Sébastien P Dion et al. also noticed a much lower expression of TMPRSS6 in HCC cells when compared to human liver samples [59]. SORD predominantly exists in liver and catalyzes the NAD+-dependent conversion of sorbitol to fructose [60]. Serum level of SORD reflects liver damage. In patients with HCC, serum levels of SORD are elevated, and serum SORD levels greater than15 ng/mL were associated with poor prognosis [61]. However, the exact role of TMPRSS6 and SORD in HCC requires further investigation. CCS is responsible for insertion of copper into Cu, Zn superoxide dismutase (SOD1), which acts as an antioxidant by eliminating toxic superoxide anion radicals [62]. Mice deficient in the SOD1 have increased oxidative stress and developed spontaneous HCC with age [63, 64], suggesting an at least indirect role of CCS in hindering the carcinogenesis of HCC. P2RX4 is an ion channel activated by extracellular ATP [65], and could also function as a pro-inflammatory receptor in cancer progression [66, 67]. Arun Asif et al. revealed that P2RX4 was significantly overexpressed in HCC [66]. And P2RX4 supports tumor growth and metastasis in other types of cancer like prostate cancer [68, 69]. ATP13A2 is a polyamine transporter which maintains healthy and functional lysosomes [70]. Although the role of ATP13A2 in HCC is currently unclear, Qian Chen et al. showed that downregulation of the gene reduced tumorigenesis of colon cancer by blocking autophagic flux [71]. LOX is a secreted copper-dependent amine oxidase, and increased level of LOX has been noted in HCC tissue, and is associated with poor prognosis of HCC patients [72, 73]. Indeed, knocking down the expression of LOX in HCC cells resulted in impaired migratory capability [73]. Taken together, HCC patients with high CMscore had high expression of P2RX4, ATP13A2 and LOX, and low expression of TMPRSS6, SORD, and CCS (Figure 2D), and might represent a more aggressive HCC subtype. Indeed, prognosis analyses revealed that HCC patients in the high-CMscore subgroup had significantly shorter OS and PFS (Figure 2H, 2J and Supplementary Figure 2A, 2C, 2D). Besides, CMscore could serve as an independent prognostic factor and had strong predictability for OS (Figures 2I, 2K, 3B, 3D). The 1-, 3-, and 5-year AUC value of CMscore revealed that CMscore exhibited a good capability in predicting prognosis of HCC patients (Figure 2I, 2K). Further, we compared the C-index of CMscore and that of some previously published signatures (Supplementary Table 6) [74–79]. Although the C-index of some signatures [75, 77] was significantly higher than that of CMscore in the TCGA-LIHC dataset, this superiority was not repeated in another dataset (GSE14520) (Supplementary Table 6). In addition, the C-index of CMscore was the highest among that of the 7 signatures in the GSE14520 dataset. Taken together, the predictability of CMscore was not inferior to other signatures. Besides, CMscore showed a significantly positive correlation with most cancer-related pathways like cell cycle process, WNT signaling and ERBB signaling (Figure 4D–4F). Indeed, ‘sustain proliferative signaling’ has been identified to be one hallmark of cancer [80]. Mitogenic signals, including ERBB signaling and WNT signaling, have been well explored in HCC in supporting its proliferative capability and the growth of tumor [81, 82]. In addition, WNT and ERBB signalings also contribute to treatment resistance and poor prognosis of HCC patients [82, 83].

Since copper participates in many biological activities, it must exert certain impact on the TME of HCC. Florida Voli et al. revealed that increased intratumor copper levels augmented the expression of PD-L1 at transcriptional and translational levels in cancer cells and facilitated cancer immune evasion. Correspondingly, copper-chelators significantly increased the level of tumor-infiltrating CD8+ T and natural killer cells [84]. However, how will copper metabolism affects TME and response to ICIs awaits more evidence. We found that CMscore had a strong positive relationship with the infiltration of most immune cells (Figure 5A–5K). In particular, HCC patients with high CMscore had significantly higher expression of pro-tumor immune infiltrates (Figure 5E) and immune checkpoint targets (Figure 6C). CMscore also showed a positive correlation with recruit of various types of immune cells, but a negative correlation with recognition of cancer cells by T cells and killing of cancer cells (Figure 6D). Taken together, HCC patients with high CMscore might exhibit an immune suppressive status. ICIs have become the keystone in the treatment for unresectable hepatocellular carcinoma [53, 85]. In two large cohorts in which cancer patients receiving ICIs, we noticed that these patients with high CMscore also exhibited high infiltration of immune cells and prolonged survival time after immunotherapy (Figure 6F–6M), suggesting cancer patients in the high CMscore subgroup might benefit from ICIs.

Further, WGCNA and subsequent correlation analyses further identified SLC27A5 exhibited a strong correlation with both CMscore (Figure 7). SLC27A5 was considerably downregulated in HCC when compared with normal tissues (Figure 8A–8D), which was consistent with previously published works [86, 87]. Xu et al. found that SLC27A5 downregulated glutathione reductase, which further led to decreased level of glutathione (GSH) [88]. Studies revealed that the depletion GSH increased sensitivity of cells to cuproptosis [23, 89], suggesting a tight relationship between SLC27A5 and cuproptosis. Since FDX1 functions as a pivotal regulator of cuproptosis [23], we investigated the relationship between SLC27A5 and FDX1 in this work. Correlation analyses in TCGA-LIHC and GSE14520 datasets, collected tissue chip, and Depmap CRISPR dataset both suggested a significant positive correlation between the expression of SLC27A5 and that of FDX1 (Figure 9A–9E). Further, upregulation of SLC27A5 indeed increase the transcriptional and protein level of FDX1 (Figure 9H, 9I). Taken together, SLC27A5 could be a potential target of HCC, partially through the induction of cuproptosis via GSH and FDX1.

At last, several limitations of this work should be pointed out. Firstly, the study predominantly relies on publicly available HCC datasets, which may have inherent limitations such as sample heterogeneity, thus, the value of CMscore in predicting prognosis of HCC patients should be validated in collected tumor samples. Secondly, high throughout RNA-seq techniques have inherent bias in evaluating the precise transcriptional level of genes. Consequently, the CMscore, based on real time polymerase chain reaction (RT-PCR) techniques, could be tested on fresh tumor samples by further studies, since this method might be more economical and convenient in clinical practice. Thirdly, the validity of CMscore in predicting response to immunotherapy should be validated by well-designed prospective clinical studies.

## CONCLUSIONS

In conclusion, HCC patients exhibited dysregulated copper metabolism. CMscore, a novel risk score based on copper metabolism-related genes, was developed using the LASSO Cox regression model. HCC patients with high CMscore were enriched in most cancer-related pathways like MAPK signaling pathway and worse prognosis. In addition, these patients also exhibited an immune suppressive status and might benefit from immunotherapy. SLC27A5, a hub gene of CMscore, might be a potential regulator of cuproptosis in HCC.

## Supplementary Material

Supplementary Figures

Supplementary Table 1

Supplementary Table 2

Supplementary Table 3

Supplementary Tables 4 and 5

Supplementary Table 6
