# Supplementary material for: Copper metabolism-related risk score identifies hepatocellular carcinoma subtypes and SLC27A5 as a potential regulator of cuproptosis
Source: Aging (Albany NY). 2023 Dec 28;15(24):15084–113. doi: 10.18632/aging.205334 (PMC10781498; doi:10.18632/aging.205334)
Supplement: Supplementary Figures [file aging-15-205334-s001.pdf]

SUPPLEMENTARY FIGURES

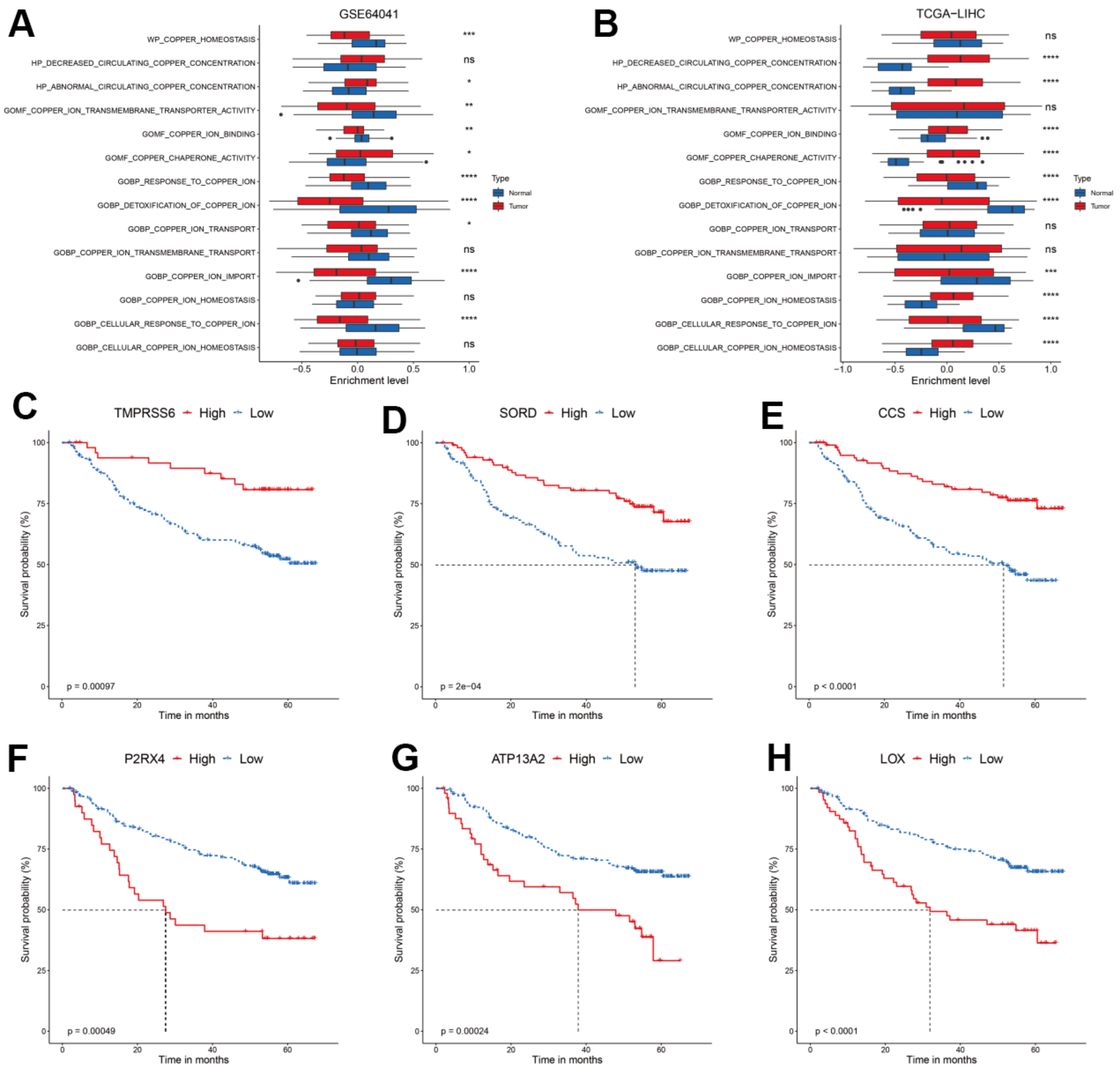

Supplementary Figure 1. Copper metabolism was aberrantly expressed in HCC and might affect prognosis of HCC patients. (**A**, **B**) The box plot showing the enrichment level of 14 copper metabolism in tumor and corresponding normal tissues from GSE64041 (**A**) and TCGA-LIHC (**B**) dataset. (**C**–**H**) Prognosis analyses of HCC patients stratified by the expression of TMPRSS6 (**C**), SORD (**D**), CCS (**E**), P2RX4 (**F**), ATP13A2 (**G**) and LOX (**H**). P-values were shown as *$p < 0.05$, **$p < 0.01$, ***$p < 0.001$, and ****$p < 0.0001$.

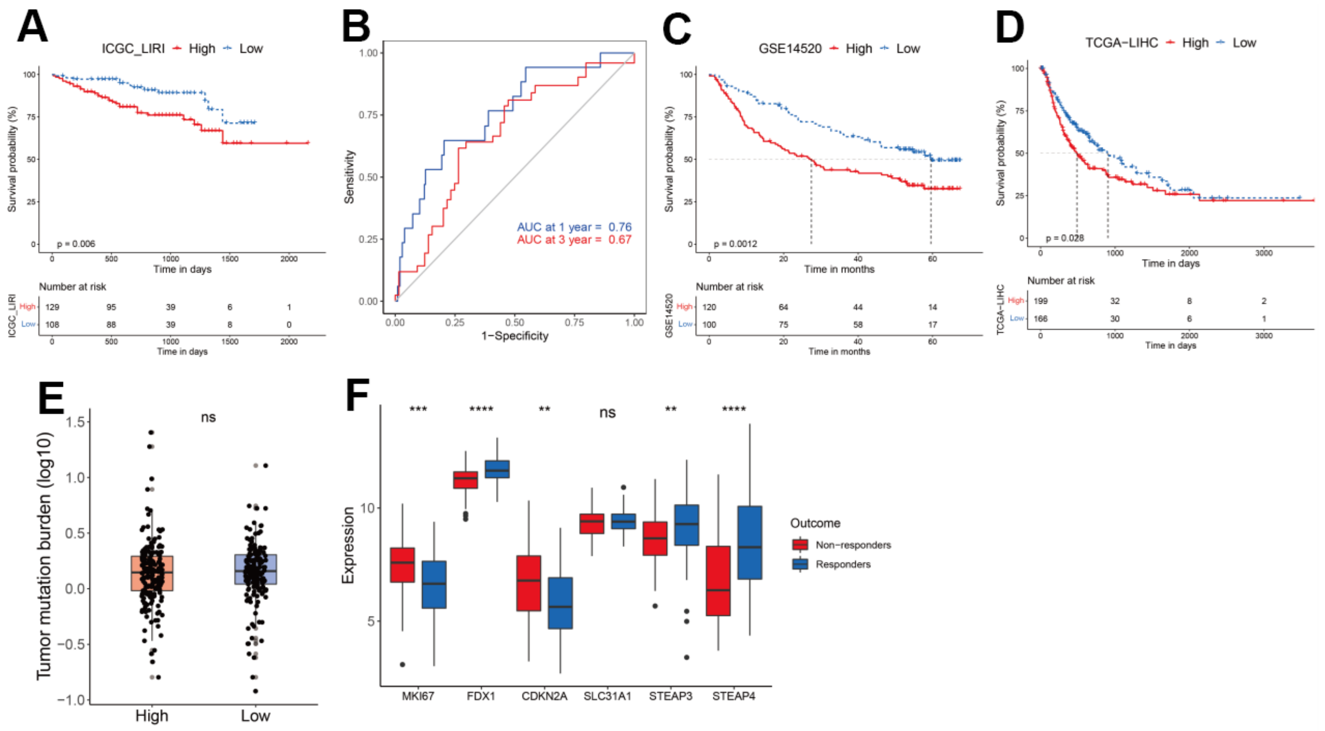

Supplementary Figure 2. Prognosis and TMB analyses in CMscore stratified HCC subgroups. (**A**) The prognostic significance of CMscore in the ICGC-LIRI dataset. The Kaplan-Meier method was used for prognosis analysis. (**B**) Time-dependent ROC analyses of the CMscore regarding the OS and survival status in the ICGC-LIRI dataset. (**C**, **D**) PFS analyses of HCC patients with high and low CMscore in the GSE14520 (**C**) and TCGA-LIHC (**D**) cohorts. (**E**) Box and dot plot showing the TMB of HCC patients from high- and low-CMscore subgroups. Wilcoxon test was used for data analyses. (**F**) The boxplot showing the expression of MKI67, cuproptosis-related genes, and copper ion import related genes between responders and non-responders to TACE.
