# Supplementary material for: Copper metabolism-related risk score identifies hepatocellular carcinoma subtypes and SLC27A5 as a potential regulator of cuproptosis
Source: Aging (Albany NY). 2023 Dec 28;15(24):15084–113. doi: 10.18632/aging.205334 (PMC10781498; doi:10.18632/aging.205334)
Supplement: Supplementary Table 1 [file aging-15-205334-s002.pdf]

SUPPLEMENTARY TABLES

Supplementary Table 1. Clinicopathological information of HCC patients whose samples were used in the tissue chip.

Demographic data		Clinical data							
Age	Gender	T	N	M	Stage	HBV infection	HCV infection	liver cirrhosis	Child-pugh
32	Female	T4	N0	M0	IIIB	HBV infection	HCV infection	Yes	A
32	Male	T2	N0	M0	II	Yes	No	Yes	A
38	Male	T2	N0	M0	II	Yes	No	Yes	A
43	Female	T2	N0	M0	II	Yes	No	No	A
45	Male	T2	N0	M0	II	Yes	No	Yes	A
46	Female	T2	N0	M0	II	Yes	No	Yes	A
47	Male	T2	N0	M0	II	Yes	No	Yes	A
47	Female	T1	N0	M0	I	Yes	No	No	A
48	Male	T2	N0	M0	II	Yes	No	Yes	A
49	Male	T2	N0	M0	II	Yes	No	Yes	A
49	Male	T3	N0	M0	IIIA	Yes	No	Yes	A
50	Male	T4	N0	M0	IIIB	Yes	No	Yes	A
52	Male	T3	N0	M0	IIIA	No	Yes	Yes	B
53	Female	T2	N0	M0	II	Yes	No	No	A
53	Female	T2	N0	M0	II	Yes	No	Yes	A
54	Male	T1	N0	M0	I	Yes	No	No	A
54	Male	T1	N0	M0	I	Yes	No	No	A
54	Female	T1	N0	M0	I	Yes	No	Yes	A
54	Male	T3	N0	M0	IIIA	Yes	No	Yes	A
54	Male	T3	N0	M0	IIIA	No	No	No	A
55	Female	T3	N0	M0	IIIA	Yes	No	No	A
55	Male	T4	N0	M0	IIIB	Yes	No	Yes	A
57	Female	T1	N0	M0	I	No	No	Yes	A
57	Female	T3	N0	M0	IIIA	Yes	No	Yes	A
59	Female	T2	N0	M0	II	Yes	No	Yes	A
59	Female	T2	N0	M0	II	Yes	No	Yes	A
59	Female	T2	N0	M0	II	Yes	No	Yes	A
59	Female	T3	N0	M0	IIIA	No	Yes	No	A
59	Male	T4	N0	M0	IIIB	Yes	No	Yes	A
61	Male	T2	N0	M0	II	Yes	No	No	A
62	Female	T4	N0	M0	IIIB	Yes	No	No	B
62	Male	T2	N0	M0	II	No	No	Yes	A
62	Male	T4	N0	M0	IIIB	No	No	Yes	A
63	Female	T2	N0	M0	II	No	No	No	A
63	Female	T2	N0	M0	II	No	No	No	A
64	Male	T1	N0	M0	I	No	No	Yes	A
65	Female	T2	N0	M0	II	Yes	No	Yes	B
66	Male	T4	N0	M0	IIIB	No	No	No	A
66	Female	T4	N0	M0	IIIB	No	No	No	A
68	Female	T1	N0	M0	I	No	No	Yes	A
69	Male	T1	N0	M0	I	No	No	Yes	A
69	Female	T1	N0	M0	I	Yes	No	Yes	A
69	Female	T1	N0	M0	I	No	Yes	Yes	A
72	Male	T4	N0	M0	IIIB	No	No	No	A

74	Female	T1	N0	M0	I	No	No	Yes	A
77	Male	T2	N0	M0	II	Yes	No	Yes	A
