# Supplementary material for: Copper metabolism-related risk score identifies hepatocellular carcinoma subtypes and SLC27A5 as a potential regulator of cuproptosis
Source: Aging (Albany NY). 2023 Dec 28;15(24):15084–113. doi: 10.18632/aging.205334 (PMC10781498; doi:10.18632/aging.205334)
Supplement: Supplementary Tables 4 and 5 [file aging-15-205334-s005.pdf]

Supplementary Table 4. GSEA analysis in HCC patients with high CMscore.

NAME	NES	NOM p-val	FDR q-val
KEGG_FC_GAMMA_R_MEDIATED_PHAGOCYTOSIS	1.8984437	0	0.17004614
KEGG_ENDOCYTOSIS	1.819987	0.002061856	0.16127017
KEGG_NOTCH_SIGNALING_PATHWAY	1.8202299	0.00210084	0.20135938
KEGG_MAPK_SIGNALING_PATHWAY	1.6682875	0.0041841	0.1557375
KEGG_GNRH_SIGNALING_PATHWAY	1.6934952	0.006085193	0.14486559
KEGG_PANCREATIC_CANCER	1.7722937	0.008097166	0.21240525
KEGG_NOD_LIKE_RECEPTOR_SIGNALING_PATHWAY	1.8361194	0.008130081	0.2266231
KEGG_PATHWAYS_IN_CANCER	1.7375147	0.010373444	0.15497063
KEGG_PATHOGENIC_ESCHERICHIA_COLI_INFECTION	1.7488441	0.011904762	0.17216738
KEGG_PURINE_METABOLISM	1.6673263	0.012170386	0.14906801
KEGG_GAP_JUNCTION	1.6483914	0.012396694	0.14366534
KEGG_BLADDER_CANCER	1.7637283	0.013435701	0.19546312
KEGG_SNARE_INTERACTIONS_IN_VESICULAR_TRANSPORT	1.7114455	0.016064256	0.16438843
KEGG_REGULATION_OF_ACTIN_CYTOSKELETON	1.7046009	0.016460905	0.15059586
KEGG_VIBRIO_CHOLERAE_INFECTION	1.7124143	0.018292682	0.17648296
KEGG_SMALL_CELL_LUNG_CANCER	1.6785665	0.018329939	0.15270367
KEGG_MELANOGENESIS	1.6630584	0.018595042	0.13967341
KEGG_AXON_GUIDANCE	1.6471983	0.02	0.12902525
KEGG_GLYCOSAMINOGLYCAN_BIOSYNTHESIS_CHONDROITIN_SULFATE	1.6451277	0.024844721	0.12586027
KEGG_BASAL_CELL_CARCINOMA	1.5684719	0.027027028	0.13928673
KEGG_EPITHELIAL_CELL_SIGNALING_IN_HELICOBACTER_PYLORI_INFECTION	1.6477886	0.027667984	0.13871863
KEGG_P53_SIGNALING_PATHWAY	1.5558062	0.028089888	0.1365577
KEGG_CHRONIC_MYELOID_LEUKEMIA	1.6473626	0.028806584	0.1338181
KEGG_CELL_CYCLE	1.7635086	0.030364372	0.1712885
KEGG_T_CELL_RECEPTOR_SIGNALING_PATHWAY	1.7456275	0.030549899	0.1598494
KEGG_WNT_SIGNALING_PATHWAY	1.5573068	0.030737706	0.13850737
KEGG_PHOSPHATIDYLINOSITOL_SIGNALING_SYSTEM	1.6307317	0.032193158	0.12288766
KEGG_TYPE_II_DIABETES_MELLITUS	1.5539867	0.032319393	0.13243896
KEGG_LONG_TERM_DEPRESSION	1.5745752	0.032388665	0.14137192
KEGG_MELANOMA	1.5366602	0.032520324	0.13525765
KEGG_LEISHMANIA_INFECTION	1.709076	0.03305785	0.15535463
KEGG_GLYCEROPHOSPHOLIPID_METABOLISM	1.4821936	0.033464566	0.14194037
KEGG_DILATED_CARDIOMYOPATHY	1.6396748	0.036072146	0.12726225
KEGG_CYTOSOLIC_DNA_SENSING_PATHWAY	1.6315686	0.0375	0.12604359
KEGG_B_CELL_RECEPTOR_SIGNALING_PATHWAY	1.697551	0.037549406	0.14886461
KEGG_CHEMOKINE_SIGNALING_PATHWAY	1.663699	0.03757829	0.14522162
KEGG_ETHER_LIPID_METABOLISM	1.5466341	0.03960396	0.13287674
KEGG_ECM_RECEPTOR_INTERACTION	1.6233107	0.04208417	0.12099782
KEGG_SPHINGOLIPID_METABOLISM	1.5098191	0.042682927	0.14172791
KEGG_FC_EPSILON_RI_SIGNALING_PATHWAY	1.6230218	0.043737575	0.117826074
KEGG_HEDGEHOG_SIGNALING_PATHWAY	1.5247976	0.046184737	0.13716604
KEGG_VEGF_SIGNALING_PATHWAY	1.5736735	0.046875	0.1385639
KEGG_CYTOKINE_CYTOKINE_RECEPTOR_INTERACTION	1.6529329	0.047131147	0.14498721
KEGG_HOMOLOGOUS_RECOMBINATION	1.567613	0.048117153	0.13666774
KEGG_TOLL_LIKE_RECEPTOR_SIGNALING_PATHWAY	1.6257775	0.048484847	0.122614816
KEGG_PRIMARY_IMMUNODEFICIENCY	1.634781	0.048484847	0.12693438

Supplementary Table 5. GSEA analysis in HCC patients with low CMscore.

NAME	NES	NOM p-val	FDR q-val
KEGG_RETINOL_METABOLISM	-2.167813	0	0.002035083
KEGG_PEROXISOME	-2.0712562	0	0.002079365
KEGG_GLYCINE_SERINE_AND_THREONINE_METABOLISM	-2.1039007	0	0.002399225
KEGG_DRUG_METABOLISM_CYTOCHROME_P450	-2.0773907	0	0.002495238
KEGG_BETA_ALANINE_METABOLISM	-2.1281288	0	0.002568046
KEGG_TRYPTOPHAN_METABOLISM	-2.0808556	0	0.002895563
KEGG_FATTY_ACID_METABOLISM	-2.0415673	0	0.003641113
KEGG_ARGININE_AND_PROLINE_METABOLISM	-1.9889241	0	0.006149278
KEGG_BUTANOATE_METABOLISM	-1.9770343	0	0.006535998
KEGG_PROPANOATE_METABOLISM	-1.9579531	0	0.006807423
KEGG_PPAR_SIGNALING_PATHWAY	-1.9671538	0	0.006864346
KEGG_DRUG_METABOLISM_OTHER_ENZYMES	-1.9174808	0	0.009751262
KEGG_VALINE_LEUCINE_AND_ISOLEUCINE_DEGRADATION	-1.9209632	0	0.010396404
KEGG_LINOLEIC_ACID_METABOLISM	-1.8366377	0	0.016201999
KEGG_PRIMARY_BILE_ACID_BIOSYNTHESIS	-1.8980548	0.002070393	0.009768278
KEGG_HISTIDINE_METABOLISM	-1.9145805	0.002109705	0.009277614
KEGG_STEROID_HORMONE_BIOSYNTHESIS	-1.911821	0.002136752	0.008890382
KEGG_TYROSINE_METABOLISM	-1.8882285	0.004175365	0.009211825
KEGG_METABOLISM_OF_XENOBIOTICS_BY_CYTOCHROME_P450	-1.893714	0.004273505	0.009375573
KEGG_LYSINE_DEGRADATION	-1.818755	0.006122449	0.018197488
KEGG_ASCORBATE_AND_ALDARATE_METABOLISM	-1.8160095	0.010351967	0.018044308
KEGG_CITRATE_CYCLE_TCA_CYCLE	-1.829712	0.012145749	0.017026087
KEGG_ALANINE_ASPARTATE_AND_GLUTAMATE_METABOLISM	-1.748662	0.012711864	0.03250989
KEGG_PYRUVATE_METABOLISM	-1.7198697	0.014799154	0.038862422
KEGG_PARKINSONS_DISEASE	-1.868609	0.029106028	0.011506476
KEGG_NITROGEN_METABOLISM	-1.5743402	0.03411514	0.08322459
KEGG_CYSTEINE_AND_METHIONINE_METABOLISM	-1.5310292	0.036734693	0.098343
KEGG_COMPLEMENT_AND_COAGULATION_CASCADES	-1.7384919	0.03877551	0.034447856
KEGG_STARCH_AND_SUCROSE_METABOLISM	-1.5690577	0.039337475	0.08343582
KEGG_GLYOXYLATE_AND_DICARBOXYLATE_METABOLISM	-1.6042256	0.039915968	0.08150707
KEGG_PENTOSE_AND_GLUCURONATE_INTERCONVERSIONS	-1.6057246	0.041841004	0.083651654
KEGG_PORPHYRIN_AND_CHLOROPHYLL_METABOLISM	-1.5906465	0.045161292	0.08498338
KEGG_BIOSYNTHESIS_OF_UNSATURATED_FATTY_ACIDS	-1.5831201	0.045738045	0.08373493
KEGG_ADIPOCYTOKINE_SIGNALING_PATHWAY	-1.5256513	0.046747968	0.096184686
KEGG_GLYCOLYSIS_GLUCONEOGENESIS	-1.5889574	0.04821803	0.08305111
KEGG_PHENYLALANINE_METABOLISM	-1.5288436	0.0491453	0.096786246
